# Immediate histologic correlation in patients with different HPV genotypes and ages: a single center analysis in China

**DOI:** 10.1186/s12885-023-11697-2

**Published:** 2023-12-08

**Authors:** Linghua Kong, Xiaoping Xiao, Tao Xu, Ru Wan, Fei Chen

**Affiliations:** 1 Department of Obstetrics and Gynecology, Peking Union Medical College Hospital, National Clinical Research Center for Obstetric & Gynecologic Diseases, Chinese Academy of Medical Sciences & Peking Union Medical College, Beijing, China; 2 Institute of Basic Medical Sciences, Chinese Academy of Medical Sciences & Peking Union Medical College, Beijing, China

**Keywords:** Human papillomavirus (HPV), Genotypes distribution, Cervical intraepithelial neoplasia (CIN), Age

## Abstract

**Background:**

Human papillomavirus (HPV) has been confirmed as a major causative factor for malignant transformation of cervical epithelial cells and for the development of cervical intraepithelial neoplasia (CIN) and invasive cervical cancer. We carried out this study to investigate the association of different HPV genotypes and ages with immediate histological cervical lesions in opportunistic screening patients in a single center.

**Methods:**

A total of 1,661 samples with biopsy-confirmed histologic findings were collected from the gynecological clinic of our hospital between October 2017 and May 2020 for analysis. The distribution of single-type HPV genotypes in CIN of different severities and the age-dependent prevalence for single-type HPV infection were analyzed.

**Results:**

In both CIN2 and CIN3 group, HPV16, 58, 52, 33 and 31/18 were detected as top 5 high-risk human papillomavirus (hrHPV) types, which accounts for 89.25% and 88.54% of single HPV infection incidence respectively. Besides, not a single case of HPV45 was found in CIN2 and CIN3. HPV16 was the dominant genotype in both CIN2 and CIN3, accounted for 46.24% and 55.21%, respectively. The prevalence of HPV16 was the most frequent in all the age groups, except ≥ 65 years group in CIN3, and almost one in three HPV16-positive patients were diagnosed with high grade CIN. The peak of the incidence of CIN3 was observed at 25 ~ 34 years (33.68%), followed by 35 ~ 44 years (31.58%).

**Conclusion:**

High grade CIN peak at 25 ~ 44 years, women of this age are recommended for normative screening if conditions permit. HPV16-positive patients should be given high priority in opportunistic screening, while the single-center data suggesting a low risk of CIN2/3 in HPV45-positive patients. For women ≥ 65 years old, patients infected with other HPV types should be also taken seriously. In general, HPV16, 58, 52, 33, 31 and 18 were the most common genotypes in CIN2/3, and a vaccine including these predominant genotypes might be of great significance for cervical cancer prevention in China.

## Background

Cervical cancer is the fourth most frequently diagnosed cancer and the fourth leading cause of cancer death among women globally, especially in developing countries. It has been estimated that there were about 604,000 new cases and 342,000 deaths worldwide in 2020 [1]. In China, there were 119,300 new cases of cervical cancer and 30,500 deaths in 2020 [2]. Human papillomavirus (HPV) is the most common sexually transmitted virus, which has been reported as the major causative factor for malignant transformation of cervical epithelial cells and for the development of cervical intraepithelial neoplasia (CIN) and invasive cervical cancer. To date, 14 HPV genotypes have been classified “high risk” due to their strong carcinogenic potential, including HPV16, 18, 31, 33, 35, 39, 45, 51, 52, 56, 58, 59, which were listed as Group 1 carcinogens by the International Agency for Research on Cancer (IARC), and HPV66 and HPV68 [3, 4]. Because of the difference between pathogenicity and various HPV genotypes, it is very important to understand the prevalence and type distribution of HPV in cervical lesions and cancer, especially in precancerous lesions. In January 2019, the World Health Organization (WHO) approved a global strategy aimed at eliminating the public health problem of cervical cancer. The strategy outlined the main goals and agreed targets to be achieved by 2030, and put the world on the track of eliminating cervical cancer [5]. In the era of widespread HPV-based primary screening and HPV-based triage of screen-detected cervical abnormalities, HPV genotyping will provide evidence for the future selection of vaccines targeting HPV types in specific regions, further aid the development of public health policy programs of eliminating cervical cancer by 2030. In China, some clinicians are still hesitant about whether young women should be tested for HPV. Although the risks of different HPV types are clear, the significance of the correlation between different HPV types and high grade squamous intraepithelial lesion (HSIL) in opportunistic screening is not yet very clear.

Therefore, this study analyzed the peak age of HSIL and the correlation between different HPV genotypes and HSIL, in order to provide clinicians with evidence-based medical data on the significance of peak age and different HPV genotypes in cervical opportunistic screening.

## Method

### The design

**Fig. 1:**
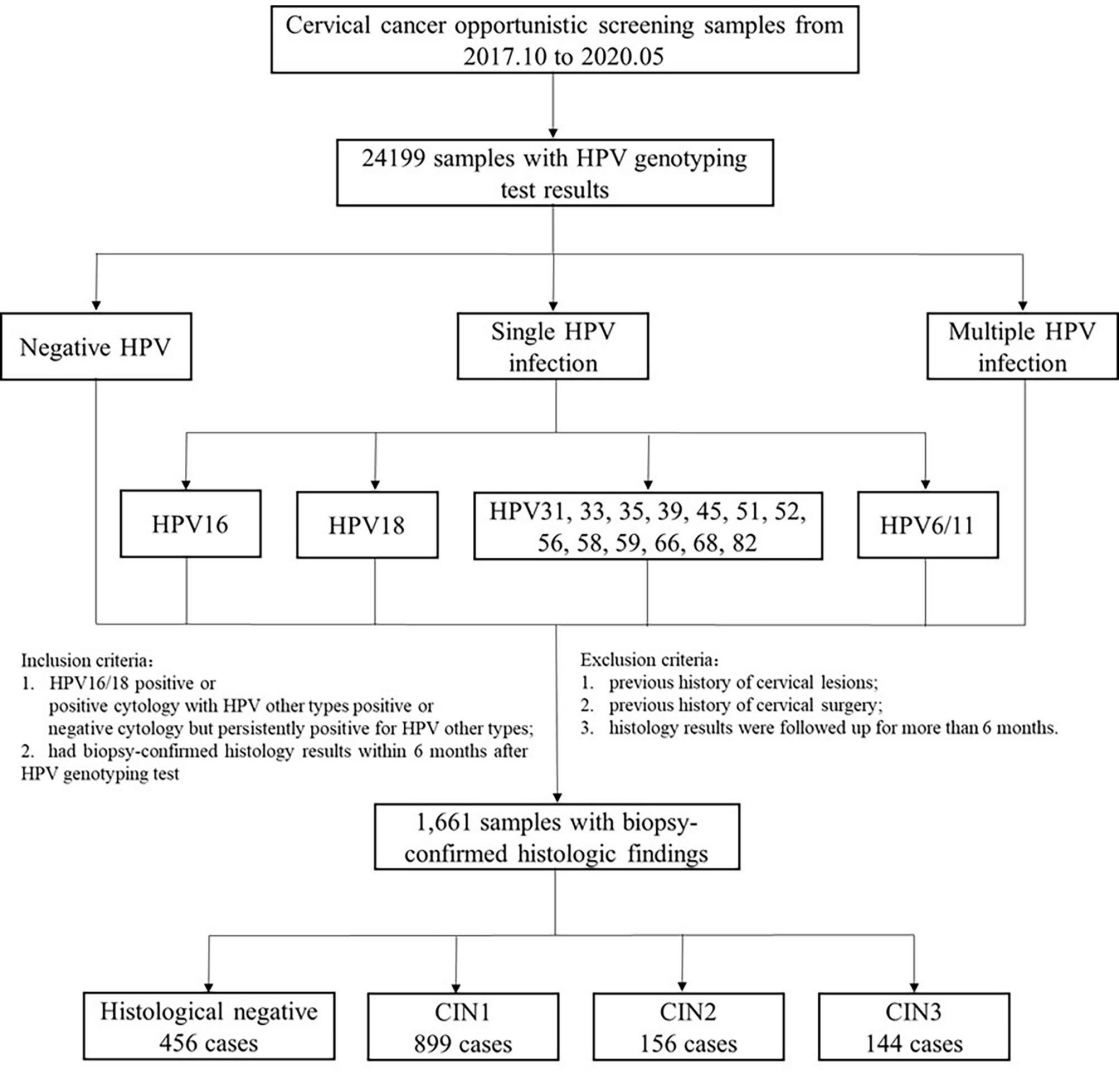
The flowchart of this study

We collected the cervical cell samples of women who visited the gynecological clinic of our hospital and accepted HPV genotyping test between October 2017 and May 2020. Inclusion criteria: (1) HPV16/18 positive or positive cytology (Atypical squamous cells of undetermined significance and above) with HPV other types positive or negative cytology but persistently positive for HPV other types (more than 1 year). (2) had biopsy-confirmed histology results within 6 months after HPV genotyping test. Exclusion criteria: (1) previous history of cervical lesions; (2) previous history of cervical surgery; (3) histology results were followed up for more than 6 months. The standard of colposcopy referral followed the 2012 ASCCP guideline and the expert consensus on colposcopy application in China [6, 7]. A total of 24,199 samples with HPV genotyping test results were collected, among which, 1,661 samples with biopsy-confirmed histologic findings were used for analysis. The mean age of the patients was 42.02 ± 11.16 years (ranged from 21 to 77 years). All biopsy-confirmed pathology were divided into 456 cases of normal, 899 cases of CIN1, 156 cases of CIN2, 144 cases of CIN3, and 6 cases of squamous cell carcinoma (SCC). We analyzed the distribution of single-type HPV genotypes in CIN of different severities and the age-dependent prevalence for single-type HPV infection (Fig. 1).

### HPV DNA test

Individual cervical samples were collected by gynecologists using a swab and placed in the preservative kit provided by the manufacturer of the HPV kit.

The HPV genotyping test was based on polymerase chain reaction (PCR) and TaqMan technology, using the commercially available HPV Genotyping Real Time PCR kit (ZJ Bio-Tech Co., Ltd., Shanghai, China), which could simultaneously detect 17 HPV types (16, 18, 31, 33, 35, 39, 45, 51, 52, 56, 58, 59, 66, 68, 82 and 6/11) [8].

### Pathological examination

Immediate histological correlation results including cervical biopsy, endocervical curettage, or loop electrosurgical excision procedure/cone biopsy performed within 6 months of HPV genotyping tests were included in this study. The histopathologic diagnoses were rendered by 2 experienced pathologists in our hospital. Histology results were categorized into negative (normal or inflammation), CIN1, CIN2, CIN3 and SCC according to the 4th edition of WHO classification of the female genital tumors [9]. CIN2+, defined as CIN2 or higher (CIN3, adenocarcinoma in situ or invasive cervical cancer). CIN3+, defined as CIN3 or higher (adenocarcinoma in situ or invasive cervical cancer).

### Statistical analysis

SPSS v25.0 was used for statistical analysis. The count data were expressed as a percentage or n (%). The prevalence of HPV across cervical lesions was compared by Chi-square test. The *P* value was used to indicate the significance, the test level was α = 0.05, and *P* < 0.05 was considered statistically significant.

## Results

**Fig. 2:**
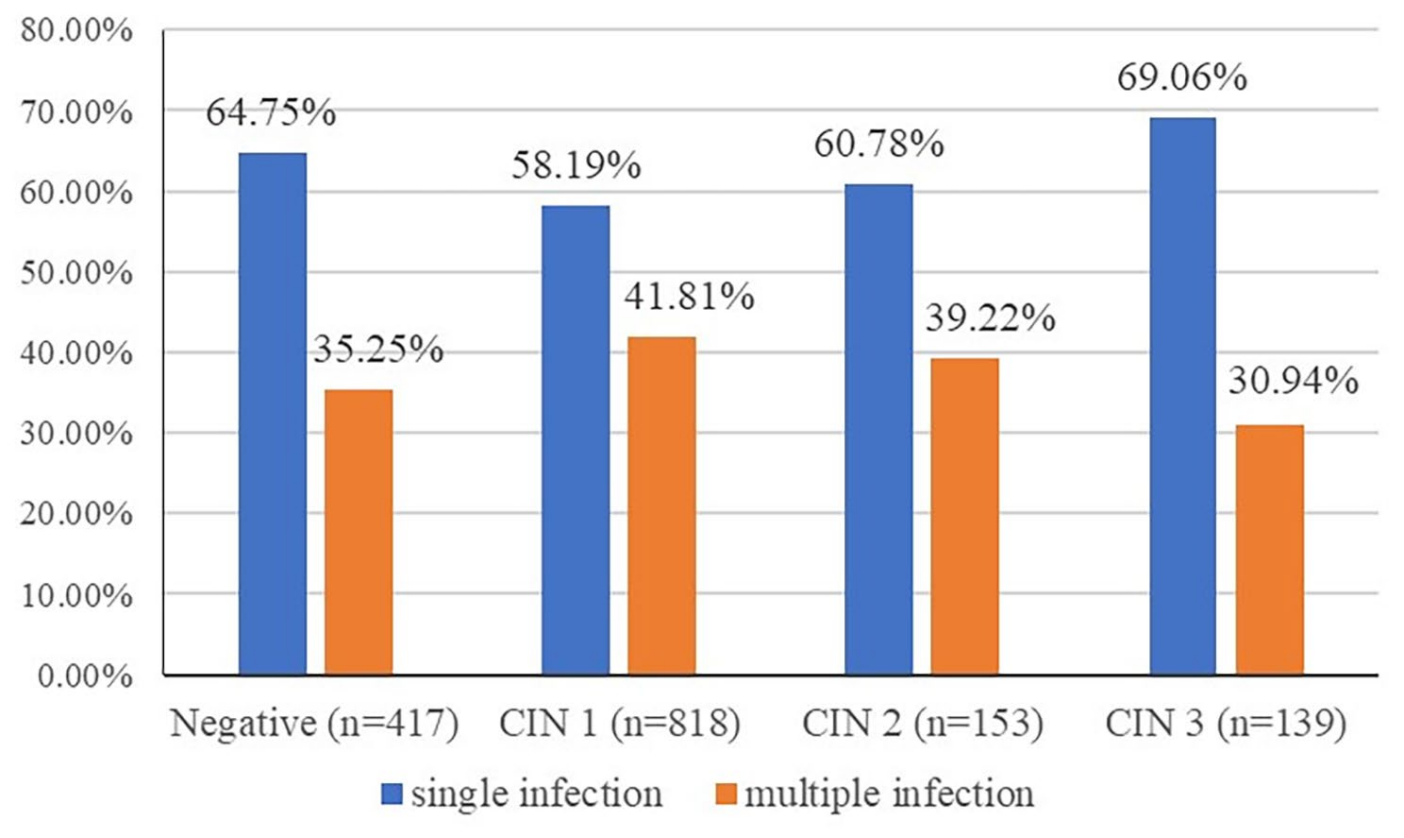
The single infection and multiple infection of HPV among different groups

A total of 24,199 samples with HPV genotyping results were collected, the overall positive rate of HPV was 23.10%. Among the 24,199 samples, 1,661 cases with histopathologic diagnoses were included in this retrospective study. 456 women were diagnosed as negative by pathology, 1,199 women were CIN, and 6 women were cervical cancer. Among the women diagnosed with CIN, 899 (75.29%) were CIN1, 156 (13.06%) were CIN2, and 144 (11.65%) were CIN3. HPV-positive results reported for negative, CIN1, CIN2, CIN3 and SCC were 91.45%, 90.99%, 98.08%, 96.53% and 100%, respectively (χ^2^ = 14.577, *P* = 0.006). As the results showed in Fig. 2, the proportion of single HPV infection increased with the increase of CIN grade, in which negative, CIN1, CIN2, CIN3 and SCC were 64.75%, 58.19%, 60.78%, 69.06%, and 100% (χ^2^ = 5.906, *P* = 0.052), respectively. However, the results for multiple HPV infection were contrary to the results for single infection and CIN grade association. The overall percentage of multiple HPV infection was 38.77% (592/1661). As shown in Fig. 2, the percentage of multiple HPV infection in women diagnosed as normal, CIN1, CIN2, CIN3 and SCC were 35.25%, 41.81%, 39.22%, 30.94% and 0%, respectively (χ^2^ = 5.906, *P* = 0.052).

### Distribution of single HPV genotypes

In this study, the evaluation values of women infected with single HPV16 type in pathologically normal CIN1, CIN2, and CIN3 were 24.07%, 22.06%, 46.24%, and 55.21%, respectively. The proportion of women infected with monotype HPV16 increased significantly with the increase of CIN grade (trend chi-square test, *P* < 0.001).

It is also observed that HPV33 and HPV16 have the same trend, that is, HPV33 infection rate increases with the increase of CIN grade. The prevalence of HPV33 in normal, CIN1, CIN2 and CIN3 were 2.22%, 3.15%, 5.38% and 8.33%, respectively (trend chi-square test, *P* = 0.033). In addition, among women with pathological normal, CIN1, CIN2 and CIN3, the incidence of HPV16/18 (HPV16 and/or HPV18) was 33.33%, 29.41%, 50.54% and 59.38% respectively (trend chi-square test, *P* < 0.001). However, the prevalence of HPV51, 56 and 66 were lower than 8.00% in all the pathological CIN grades and decreased obviously with the increasing grade of CIN (chi-squared test for trend, *P* < 0.05). Then, in normal pathology, CIN1, CIN2, and CIN3, the incidence of other high-risk human papillomavirus (hrHPV) types (excluding 16 and 18) was 5.93%, 69.54%, 49.46%, and 40.63%, respectively. In conclusion, with the increase of CIN grade, the percentage of non-HPV16/18 of hrHPV also showed the same downward trend (Chi-square test of trend, *P* < 0.001).

In women with normal pathology, the total incidence rate of the five most common genotypes (HPV16, 52, 58, 18 and 51/56, with the frequency decreasing from 24.07 to 5.19%) was 67.41%. In CIN1 patients, the 5 most common HPV types were HPV16 (22.06%), HPV52 (17.02%), HPV58 (14.92%), HPV18 (7.35%) and HPV66 (7.14%), with a total incidence of 68.49%. The total incidence rate of five major hrHPV genotypes in CIN2 women was 89.25%, which was HPV16 (46.24%), HPV52 (15.05%), HPV58 (15.0%), HPV31 (7.53%), and HPV33 (5.38%), respectively. In the CIN3 group, the infection rate of the first five hrHPV genotypes accounted for 88.54%, and the prevalence rate was HPV16 (55.21%), HPV58 (11.46%), HPV52 (9.38%), HPV33 (8.33%), HPV31/18 (4.17%), respectively. Moreover, no monotypic infection of HPV45 and HPV6/11 was observed in CIN2 and CIN3 in this study. In addition, all of the six SCC cases were detected with single HPV16 genotype results. (Shown in Table 1)

**Table 1:** Distribution of HPV genotypes in CIN

HPV types	Negative (n = 270)	CIN1 (n = 476)	CIN2 (n = 93)	CIN3 (n = 96)	*P*-value
16	24.07%	22.06%	46.24%	55.21%	< 0.001
18	9.26%	7.35%	4.30%	4.17%	0.243
31	3.70%	2.10%	7.53%	4.17%	0.053
33	2.22%	3.15%	5.38%	8.33%	0.033
35	1.48%	2.10%	1.08%	2.08%	0.871
39	4.81%	2.94%	1.08%	0.00%	0.065
45	1.48%	0.63%	0.00%	0.00%	0.325
51	5.19%	5.67%	1.08%	0.00%	0.031
52	14.81%	17.02%	15.05%	9.38%	0.295
56	5.19%	5.04%	0.00%	1.04%	0.046
58	14.07%	14.92%	15.05%	11.46%	0.842
59	2.96%	3.36%	2.15%	1.04%	0.630
66	4.81%	7.14%	1.08%	0.00%	0.006
68	4.81%	4.62%	0.00%	1.04%	0.065
82	0.37%	0.84%	0.00%	2.08%	0.311
6 + 11	0.74%	1.05%	0.00%	0.00%	0.571
16/18	33.33%	29.41%	50.54%	59.38%	< 0.001
Others*	65.93%	69.54%	49.46%	40.63%	< 0.001

*Others: HPV types includes 31, 33, 35, 39, 45, 51, 52, 56, 58, 59, 66, 68, 82. HPV: Human papillomavirus; CIN: cervical intraepithelial neoplasia

.

### Distribution patten of predominant HPV genotypes

**Fig. 3:**
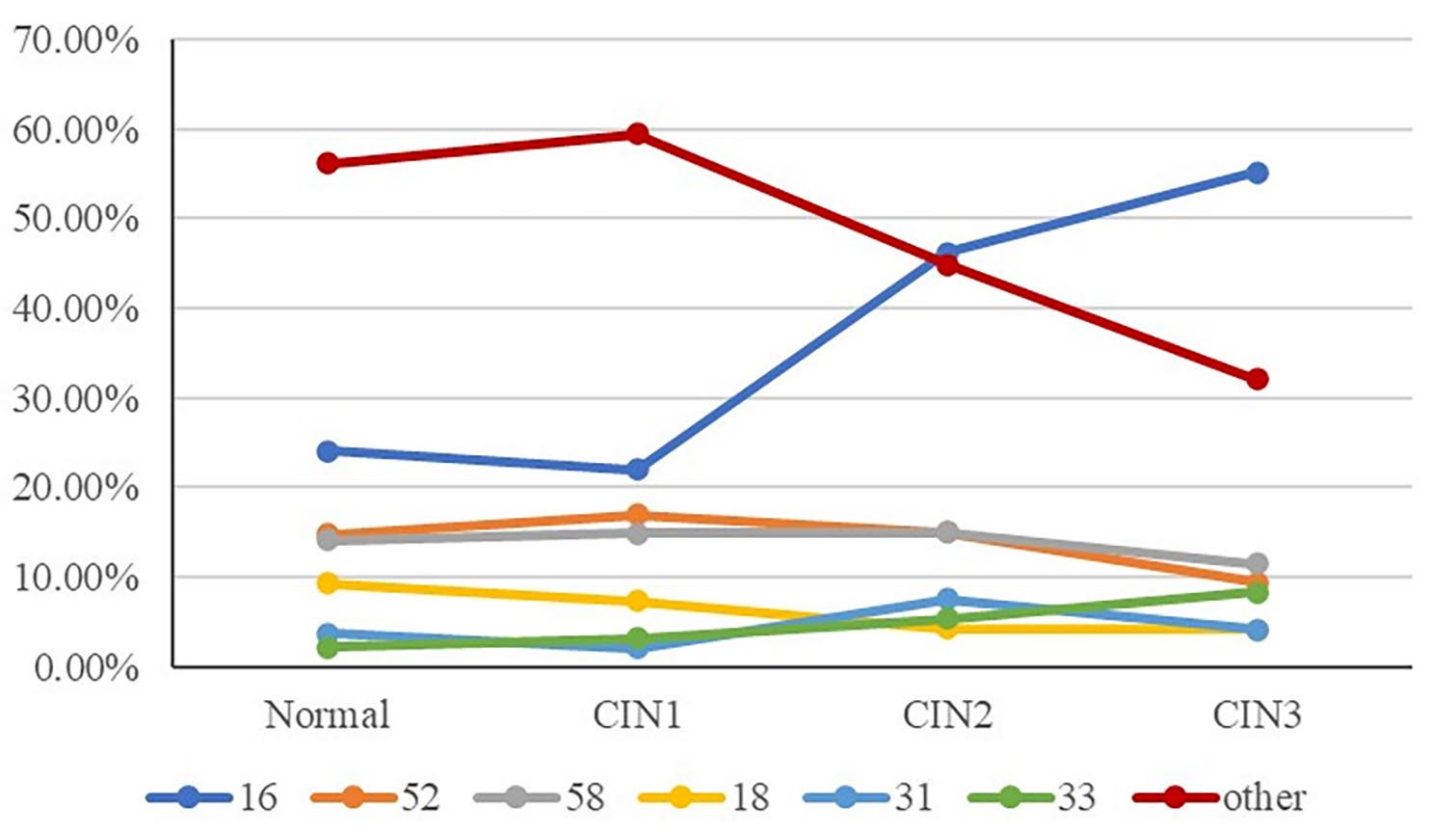
The distribution pattern of predominant HPV genotypes with single-type infection

As shown in Fig. 3, HPV16 was the most common genotype in monotypic HPV infection, and its prevalence increases significantly with the severity of CIN (Chi-square test of trend, *P* < 0.001). Even though the prevalence of HPV33 in all CIN grades was lower than 10%, HPV33 also increased with the increase of CIN grade (Chi-square test of trend, *P* = 0.033). However, HPV18, 31, 52, 58 showed the opposite trends, which decreased with increasing grade of CIN. Besides, other HPV types also decreased with increasing grade of CIN (chi-squared test for trend, *P* < 0.001). Regarding single infection, the cumulative positive rate of HPV16, 18, 31, 33, 52 and 58 was 68.15%, 66.60%, 93.55% and 92.71% in pathologic normal group, CIN1, CIN2 and CIN3, respectively.

### Coloscopy referral number for detecting 1 CIN2+/CIN3

To detect one CIN2 + cases, 2.8, 8.5, 3.2 and 7.1 women should receive colposcopy referrals if using HPV16, HPV18, HPV16/18 and non-HPV16/18, HPV others as screening methods for detecting cervical lesions, respectively. Meanwhile, to detect one CIN3 case, the referral numbers for HPV16, HPV18, HPV16/18, and HPV others were 5.0, 17.0, 5.9 and 15.4 women, respectively. The colposcopy referral rate for HPV others is 2.5 times and 3 times higher than HPV16 for detecting one CIN2 + case and one CIN3 case, respectively (Table 2).

**Table 2:** Colposcopy referral number for detecting one CIN2 + and CIN3 by using different HPV genotypes

HPV type	Negative % (n/N)	CIN1 % (n/N)	CIN2 % (n/N)	CIN3 % (n/N)	Coloscopy referral number for detecting 1 CIN2+	Coloscopy referral number for detecting 1 CIN3
HPV16 (n = 266)	24.07% (65/270)	22.06% (105/476)	46.24% (43/93)	55.21% (53/96)	2.8	5.0
HPV18 (n = 68)	9.26% (25/270)	7.35% (35/476)	4.30% (4/93)	4.17% (4/96)	8.5	17.0
HPV16/18 (n = 334)	33.33% (90/270)	29.41% (140/476)	49.46% (46/93)	59.37% (57/96)	3.2	5.9
HPV others (n = 601)	66.67% (180/270)	70.59% (336/476)	49.46% (46/93)	40.62% (39/96)	7.1	15.4

HPV: Human papillomavirus; CIN: cervical intraepithelial neoplasia

### Age-dependent prevalence for single-type HPV Infection

**Fig. 4:**
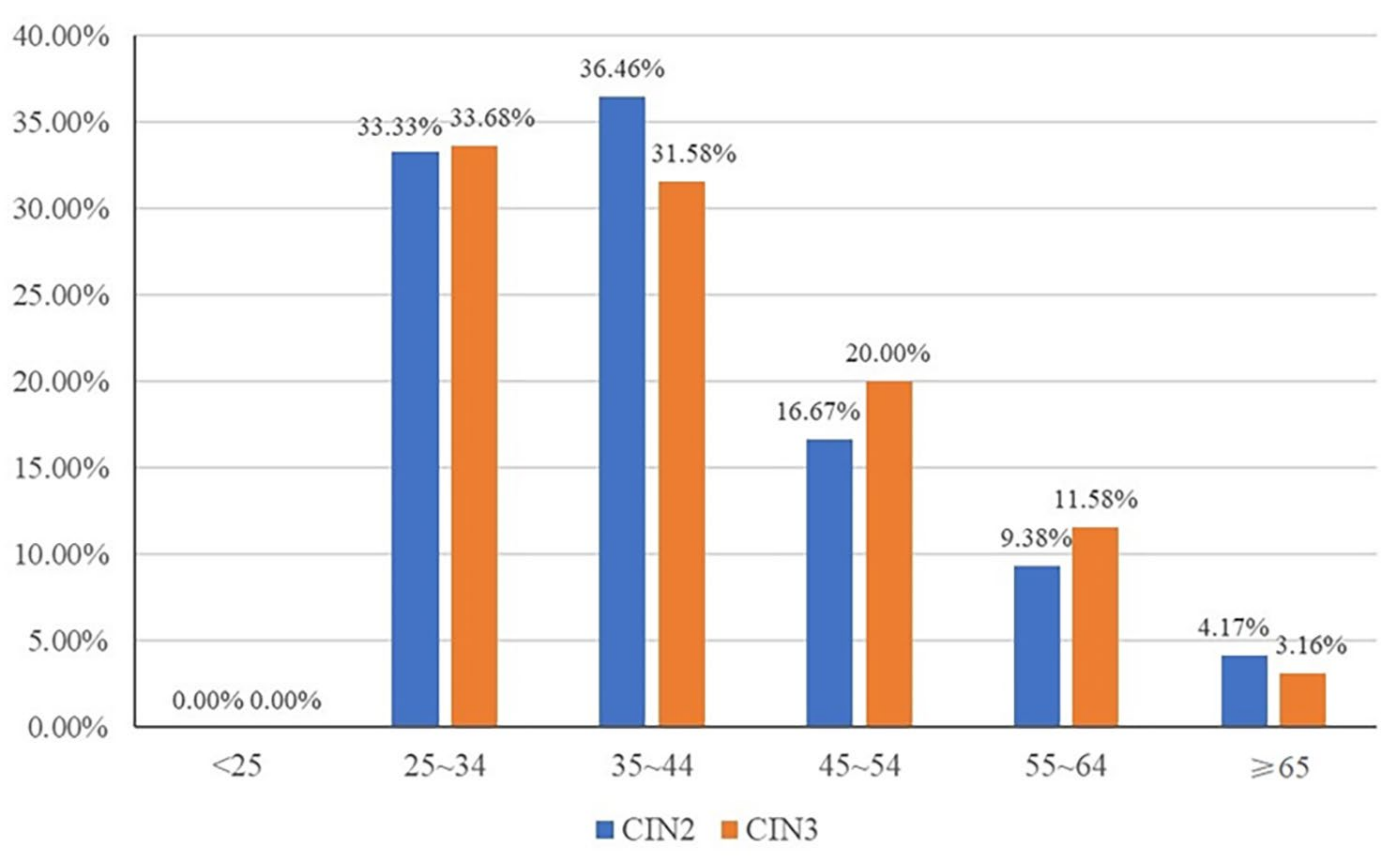
The age distribution of the incidence of CIN2 and CIN3

In the present study, the average age of CIN2 and CIN3 were (41.51 ± 10.53) years and (40.75 ± 10.51) years, respectively. In CIN2, the main age was 35 ~ 44 years (36.46%) and 25 ~ 34 years (33.33%), and the prevalence of CIN2 decreased obviously with increasing age in women over 45 years old. While the peak of the incidence of CIN3 was observed at 25 ~ 34 years (33.68%), followed by 35 ~ 44 years (31.58%), and decreased obviously with increasing age. Moreover, no single type infection was identified under 25 years old in both CIN2 and CIN3 (Fig. 4).

**Fig. 5:**
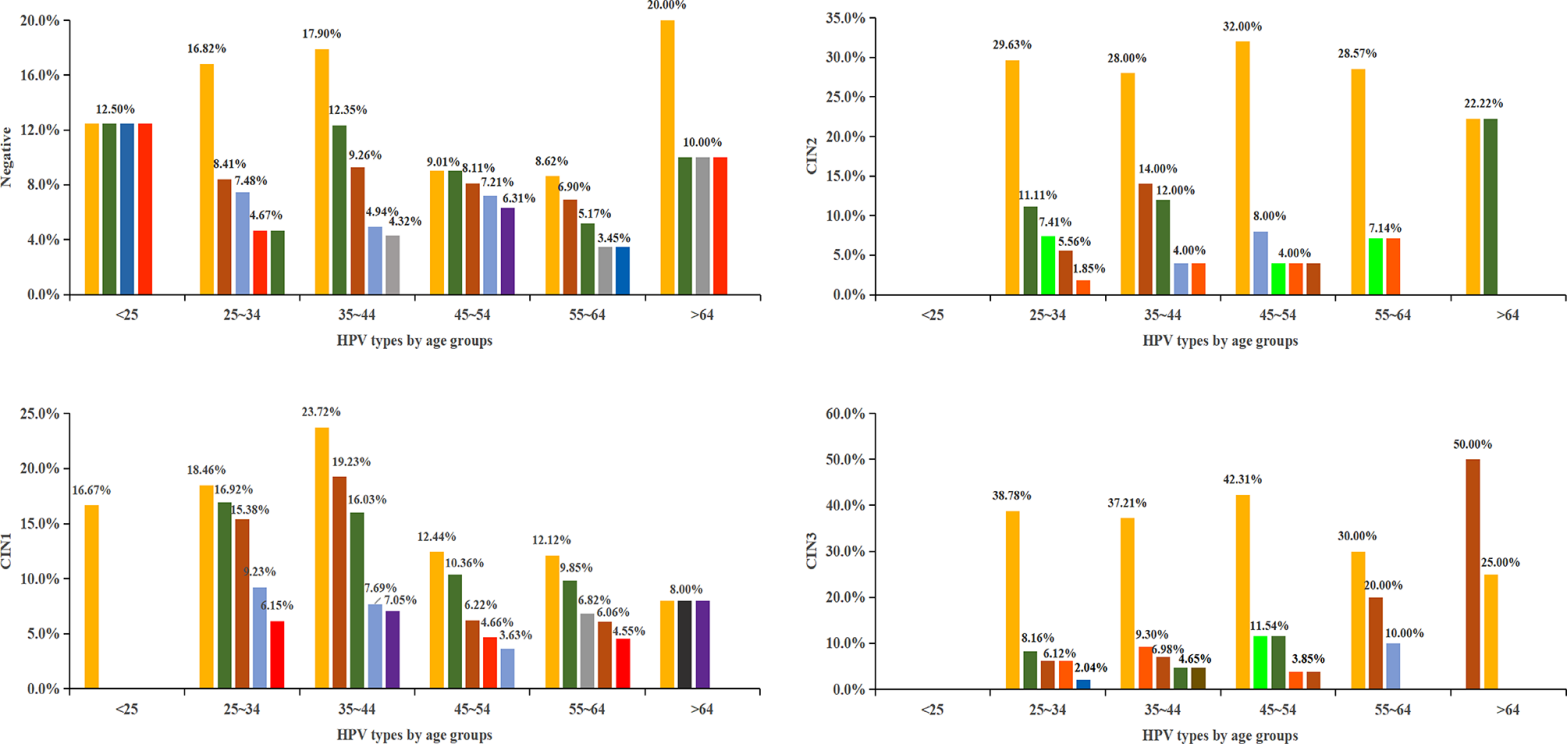
HPV genotypes by age groups in CIN

When age-dependent prevalence for single-type HPV infection in negative, CIN1, CIN2 and CIN3 were evaluated, the top 5 most frequent HPV types in different age groups were analyzed and showed in Fig. 5. In general, the prevalence of HPV16 was the most frequent in all the age groups, except ≥ 65 years group in CIN3, in which two out of three patients were HPV58 infected and one was HPV16 infected. Among CIN1 patients, HPV16, 52 and 58 were the common predominant HPV types in all the age groups except ≥ 65 years. In CIN2 patients, the most prevalent HPV types in different age groups were HPV16, 52, 58, 33, 31 and 18, with relative proportion differed somewhat by age. In CIN3 patients, HPV16, 52, 58 and 33 were the common frequent types in the age groups of 25 ~ 34 years, 35 ~ 44 years and 45 ~ 54 years. Moreover, the prevalence of HPV16 in younger groups (25 ~ 34 years, 35 ~ 44 years and 45 ~ 54 years) was significantly higher than older groups (55 ~ 64 years and ≥ 65 years), while HPV58 showed the opposite trends.

## Discussion

In the present study, the overall HPV-positive rate of the opportunistic screening was 23.10%, and the HPV-positive rate reported in the negative pathology, CIN1, CIN2, and CIN3, were 91.45%, 90.99%, 98.08% and 96.53%, which were higher than the positive rate in cervical cancer screening [10, 11]. In addition, the proportions of multiple HPV infection were 35.25%, 41.81%, 39.22%, 30.94% and 0% in negative, CIN1, CIN2, CIN3 and SCC, respectively, which decreased with increasing grade of CIN. Thus, a negative relationship between multiple infection and the progression of CIN was found, which revealed that multiple infection was not the leading factor for the progression of CIN2+, the finding was consistent with previous study [12–14]. The overall percentage of multiple HPV infection was 38.77%, which was higher than most of the domestic and foreign data [8, 15–17]. The main reason for the difference was that this study was a retrospective analysis based on opportunistic screening, the included outpatients were mostly patients from all over the country with abnormal cervical cancer screening results, so the positive rate of HPV and the proportion of multiple infection were relatively high.

When evaluating cases with single-type HPV infection, HPV16 and 33 increased significantly with the increase of CIN grade, while HPV18, 31, 52 and 58 showed the opposite trend. Besides, all of the six SCC cases in this study were infected with single-type HPV16. These results show that HPV16 was the most aggressive hrHPV in the development of cervical precancerous lesions and malignant cancer. Compared with other HPV genotypes, HPV16-infected cervical lesions were less likely to regress, which was consistent with previous studies [18, 19]. Moreover, the frequency of HPV33 also increased with the severity of the cervical lesion grade, this result was consistent with the trend reported in the previous literature [20]. This study found that Chinese women infected with HPV33 should also be concerned, and it is worth further clinical research because the cases in this analysis were limited. However, the present study demonstrated that the prevalence of HPV18 was less than 10% and decreased with the severity of cervical lesions, which was not the most common HPV genotype in CIN2/3. The finding was similar with other Chinese studies [21], but was different with international data [22, 23]. Furthermore, only 7 cases (0.94%) with single-type HPV45 were found in ≤ CIN1 patients, while no cases of single-type HPV45 were identified in both CIN2 and CIN3 in this study. The low incidence of HPV45 was consistent with data from most domestic studies [21]. Although HPV18 and 45 showed a relative lower prevalence in the current analysis, they were reported to be associated with glandular lesions in the endocervical canal [24, 25], which are still of great importance. Consequently, patients with HPV18/45 infection should pay close attention on endocervical canal lesions when referring for a coloscopy. This result suggested that the risk of CIN2/3 in HPV45-positive patients was not high based on single-center data. In addition, the referrals and colposcopies number were as low as 2.8 and 5.0 if using HPV16 as screening method for detecting one CIN2 + and one CIN3, respectively, which is much lower than that of HPV others. Therefore, HPV16-positive patients should be given high priority in opportunistic screening because almost one in three HPV16-positive patients would likely to be diagnosed with CIN2/3 in the opportunistic screening. In our hospital, gynecologists should pay more attention to the follow-up of HPV16-positive patients. It is worth mentioning that the high aggressiveness of HPV16 may be related to the genetic interaction with patients. Mogge Hajiesmaeil et al. found that Methylenetetrahydrofolate reductase (MTHFR) gene polymorphisms, including C677T and A1298C, are strongly associated with the risk of cervical cancer, it seems that MTHFR 1298CC genotype is more susceptible to HPV16 infection [26]. To better understand the high pathogenicity of HPV16, more basic research on the carcinogenicity of HPV16 is needed. Meanwhile, the data of HPV others showed in Table 2 was actually not using HPV others alone as screening method, but in combination with the results of cotest (hrHPV test and cytology). Therefore, the actual referrals and colposcopies number using HPV others alone as screening method would be higher. The frequency of colposcopy referral suggested that HPV16 has higher risk of CIN2/3 than HPV other types.

HPV genotype distribution in high-grade cervical lesions has been reported to vary significantly in different geographic population [27, 28]. According to a meta-analysis, the prevalent HPV types in high-grade cervical lesions were 16 (57.90%), 31 (15.80%), 33 (4.40%), 18 (4.00%) and 52 (2.90%) in Europe, while the top five HPV types in CIN3 were 16, 31, 18, 52 and 59 in Canada [29]. In this study, the 5 predominant genotypes were 16 (46.24%), 52 (15.05%), 58 (15.05%), 31 (7.53%) and 33 (5.38%) in CIN2 patients and the most prevalent HPV genotypes among CIN3 patients were HPV16 (55.21%), HPV58 (11.46%), HPV52 (9.38%), HPV33 (8.33%), HPV31/18 (4.17%). The prevalence of these predominant genotypes comprised 89.25% and 88.54% of total single HPV infection in CIN2 and CIN3, respectively. It is worth mentioning that most of the patients in our gynecological clinic came from all over China with confirmed HPV infection or suspected cervical lesions, which does not belong to the category of regional cervical cancer screening, but opportunistic screening. Our finding was consistent with a previous analysis [30], which reported the predominant types of CIN2/3 in Asia were HPV16, 58, 52, 18, 33 and 31. Regarding the distribution of HPV genotypes in China, little regional differences among high-grade cervical lesions were observed. In northern China, the most prevalent HPV genotypes were found to be HPV16, 58, 33, 52 and 18 [21]. In a large cohort study based on western Chinese women, the most commonly detected HPV genotypes in CIN2/CIN3 cases were HPV16 (48.1%), 58 (19.3%), 52 (10.0%), 33 (9.6%) and 18 (4.6%) [13]. In eastern China, the top 5 predominant genotypes in CIN2 were 16, 58, 52, 33 and 31, while in CIN3 were HPV16, 58, 33, 52 and 31 [12]. Thus, based on the prior studies and the current study, HPV16, 58, 52, 33, 31 and 18 were the predominant genotypes in the majority of Chinese women. Therefore, vaccine including HPV16, 18, 31, 33, 52 and 58 is potentially very effective for Chinese women, which might reduce the morbidity of cervical cancer in China. Of cause, large multicenter studies and long-term follow-up are needed to further confirm this hypothesis.

In the present study, the incidence of high-grade cervical lesions was significantly higher among women age 25 ~ 34 years and 35 ~ 44 years than among the other age groups, the results were slightly different from previous studies. In the previous population-based study in Beijing, the peak age of CIN2 + was 30 ~ 34 years [31]. Besides, a cross-sectional study of the Yangtze River Delta area (China) showed that the prevalence of CIN2 and CIN3 peaked at 40 ~ 44 years and 35 ~ 39 years, respectively, and followed by 35 ~ 39 years and 30 ~ 34 years, respectively [12]. In our study, women over 25 years old were recommended for a standardized screening when conditions permit. Besides, for younger patients under 25 years old, 6 cases of CIN2/3 infected with multiple HPV infection were found, of which, 4 cases were infected with HPV16-containing multiple infection, the other 2 cases were infected with HPV others multiple infection. The effect of having HPV infections without Pap-based care until age 25 on the prevalence of CIN 2 + and their determinants are largely unknown [32, 33]. Therefore, from the process of HPV infection to the occurrence of high-grade cervical lesions, the screening of patients younger than 25 years old should also be paid attention. When age-dependent prevalence for single-type infection was examined in CIN3 groups, we found that the prevalence of HPV16 was significantly lower in the patients older than 55 years old. In contrast, the prevalence of HPV58 was obviously higher in the patients over 55 years old. This data suggested that HPV16 is the most malignant HPV type, which has a strong potential for CIN progression. And this progression, from benign to premalignant (or malignant lesions), is earlier than that of other types. However, other HPV types, such as HPV58, may need longer time to progress into premalignant lesions. In this study, 3 cases of CIN3 were found in ≥ 65 years group, with 2 cases infected with HPV58 and one case infected with HPV16. As a country with vast territory, China still faces many difficulties in cervical cancer screening, many women ≥ 65 years old have not yet received routine cervical cancer screening before. Therefore, women ≥ 65 years old with other HPV types of infection should also receive special attention.

## Conclusion

In summary, age-dependent distribution suggested that high grade CIN peaks at 25 ~ 44 years of age, women aged 25 years and older in China are recommended for a routine screening if conditions permit. For women ≥ 65 years old, infection with other HPV types should also be taken seriously. For women at 25 to 64 years old, HPV16 was particularly aggressive in the development of cervical premalignant lesions and malignant lesions. In the single-center data, HPV16-positive patients should be given high priority in opportunistic screening because almost one in three HPV16-positive patients would likely to be diagnosed with CIN2/3 in the opportunistic screening, while HPV45 suggested low risk of CIN2/3 in the single-center study. In general, HPV16, 58, 52, 33, 31 and 18 were the most common genotypes in high grade CIN, vaccines including these main genotypes might be of great value for cervical cancer prevention in China. The current study was a single-center and retrospective analysis with a relatively small sample size, further confirmation and validation are needed in future multi-center prospective studies with large samples to provide data for opportunistic screening.

## Data Availability

All data generated or analyzed during this study are included in this published article.

## List of abbreviations

HPV: Human papillomavirus
hrHPV: High-risk human papillomavirus
CIN: Cervical intraepithelial neoplasia
HSIL: High grade squamous intraepithelial lesion
SCC: Squamous cell carcinoma
PCR: Polymerase chain reaction
